# Recessive Variants in *PIGG* Cause a Motor Neuropathy with Variable Conduction Block, Childhood Tremor, and Febrile Seizures: Expanding the Phenotype

**DOI:** 10.1002/ana.27113

**Published:** 2024-10-23

**Authors:** Christopher J. Record, Antoinette O'Connor, Nienke E. Verbeek, Wouter van Rheenen, Eleni Zamba Papanicolaou, Stojan Peric, Peter C. Ligthart, Mariola Skorupinska, Ellen van Binsbergen, Philippe M. Campeau, Vukan Ivanovic, Brian Hennigan, John C. McHugh, Julian C. Blake, Yoshiko Murakami, Matilde Laura, Sinéad M. Murphy, Mary M. Reilly

**Affiliations:** ^1^ Centre for Neuromuscular Diseases, Department of Neuromuscular Diseases UCL Queen Square Institute of Neurology London UK; ^2^ Department of Neurology Tallaght University Hospital Dublin Ireland; ^3^ Department of Genetics University Medical Centre Utrecht Utrecht The Netherlands; ^4^ Department of Neurology UMC Utrecht Brain Center, University Medical Center Utrecht, Utrecht University Utrecht The Netherlands; ^5^ Neuroepidemiology Department of The Cyprus Institute of Neurology and Genetics Nicosia Cyprus; ^6^ Faculty of Medicine, University of Belgrade Belgrade Serbia; ^7^ Neurology Clinic, University Clinical Centre of Serbia Belgrade Serbia; ^8^ Department of Immunohematology Diagnostic Services Sanquin Diagnostic Services Amsterdam The Netherlands; ^9^ Department of Pediatrics University of Montreal Montreal Canada; ^10^ Clinical Neurophysiology Department Tallaght University Hospital Dublin Ireland; ^11^ Clinical Neurophysiology Department Children's Health Ireland at Crumlin Dublin Ireland; ^12^ Department of Clinical Neurophysiology Norfolk and Norwich University Hospital Norwich UK; ^13^ Laboratory of Immunoglycobiology, Research Institute for Microbial Diseases, Osaka University Suita Japan; ^14^ Academic Unit of Neurology, Trinity College Dublin Dublin Ireland

## Abstract

Biallelic variants in phosphatidylinositol glycan anchor biosynthesis, class G (*PIGG*) cause hypotonia, intellectual disability, seizures, and cerebellar features. We present 8 patients from 6 families with a childhood‐onset motor neuropathy and neurophysiology demonstrating variable motor conduction block and temporal dispersion. All individuals had a childhood onset tremor, 5 of 8 had cerebellar involvement, and 6 of 8 had childhood febrile seizures. All individuals have biallelic *PIGG* variants, including the previously reported pathogenic variant Trp505*, plus 6 novel variants. Null enzyme activity is demonstrated via *PIGO*/*PIGG* double knockout system for Val339Gly and Gly19Glu, and residual activity for Trp505* due to read‐through. Emm negative blood group status was confirmed in 1 family. *PIGG* should be considered in unsolved motor neuropathy. ANN NEUROL 2025;97:388–396

Phosphatidylinositol glycan anchor biosynthesis, class G (*PIGG*) is one of 22 phosphatidylinositol glycan (PIG) genes involved in the biosynthesis of glycosylphosphatidylinositol (GPI). GPI is a glycolipid that anchors over 150 proteins to the cell membrane, which in turn play a critical role in neurogenesis.^1^ The core glycan structure of the glycolipid GPI consists of 3 mannoses, all modified with ethanolamine‐phosphate (EtN‐P). *PIGG* is responsible for the enzyme facilitating addition of EtN‐P to the second mannose. Contrary to previous understanding that the EtN‐P on the third mannose was the bridge to the GPI‐anchored protein (GPI‐AP), it has recently been shown that selected GPI‐APs are bound by EtN‐P on the second mannose, which sheds mechanistic insight into how variants in *PIGG* might cause disease. Biallelic variants in PIG genes cause inherited GPI deficiency (IGD); a group of disorders associated with intellectual disability (ID), seizures, and facial dysmorphism.^2^ These features, in addition to cerebellar atrophy with associated ataxia and nystagmus, have recently been reported with recessive *PIGG* variants.^3^ The red blood cell (RBC) antigen Emm, which was unidentified until recently, has been proven to be free GPI,^4^ and biallelic *PIGG* variants independently identified as causing Emm‐negative blood group, with or without an associated neurodevelopmental syndrome.^5^ We describe 8 cases from 6 families carrying biallelic variants in *PIGG*, displaying a distinct neuropathy syndrome, expanding the known phenotype.

## Methods

### Patient Selection and Genetic Testing

Families were recruited in the United Kingdom, Ireland, Cyprus, The Netherlands, and Serbia with informed consent obtained from all patients according to local institutional requirements. Patients were clinically assessed by neuromuscular/neurogenetic experts. Genetic testing was performed with either whole exome sequencing (WES) or whole genome sequencing (WGS), on a clinical or research basis in line with local practice (Supplementary Table S1). Virtual panels were applied to WES/WGS data to exclude known causes of neuropathy and ID, if applicable. Variants were classified according to the American College of Medical Genetics and Genomics (ACMG) criteria.^6^

### Functional Analysis of PIGG Variants

As previously reported, *PIGO* knockout (*PIGO* KO) cells show partial loss of surface GPI‐APs, which is completely removed by further knockout of *PIGG*.^7^ Introducing the *PIGG* gene into *PIGO/PIGG* double knockout (DKO) cells restores the expression of GPI‐APs to the level of a *PIGO* single knockout, allowing the activity of *PIGG* variants to be analyzed by flow cytometry. It has previously been shown that decay‐accelerating factor (DAF) and cell surface Fc receptor CD16 are sensitive to the partially reduced activity of PIGG. Therefore, to measure the *PIGG* variant activity, *PIGO/PIGG* DKO HEK293 cells (permanently expressing CD16) were transfected with the strong SRα promoter (pME) or weaker thymidine kinase promoter (pTK) driven wild‐type (WT) or mutant *PIGG*‐glutathione S‐transferase (*PIGG‐*GST) plasmids.^3^ To determine transfection efficiency, luciferase expression plasmid was co‐transfected with *PIGG*‐GST plasmids. Restoration of the surface expression of DAF and CD16 was analyzed 2 days later by staining cells with anti‐DAF antibody (clone IA10) or anti‐CD16 antibody (3G8 Biolegend) followed by phycoerythrin labeled anti‐mouse IgG and analyzed by flow cytometry. An isotype control antibody, matching the class of the test antibody but not targeting any antigen (DAF, mouse IgG2a; CD16, and mouse IgG1), was used to confirm fluorescence is due to specific antibody binding. The protein expression of each *PIGG*‐GST variant was then analyzed by Western blotting with anti‐GST antibody (anti‐goat GST; GE Healthcare) using the cell lysate of each transfectant. To quantify PIGG‐GST levels, band intensities of PIGG‐GST were divided by the band intensities of GAPDH (loading control) and by luciferase activities (transfection efficiency).

### Emm Blood Group Testing

Emm blood group antigen testing was performed as previously described.^5^ The presence of the red cell Emm antigen was determined with a hemagglutination assay (indirect antiglobulin test in tubes with polyethylene glycol as enhancer). The anti‐Emm used was a polyclonal human antiserum, with anti‐Emm antibodies of the IgG class and produced by an unrelated Emm negative female patient.

## Results

### Clinical Description

Eight patients from 6 non‐consanguineous families were identified with biallelic variants in *PIGG*. Clinical characteristics are summarized in Table 1 and Supplementary Table S1. Two of 8 patients were male with mean age at assessment of 28.3 years. The age of onset of neuromuscular symptoms ranged from 4 months to early teens; all but one individual presented with lower limb symptoms. All had a postural tremor (mean age of onset 7.1 years, Supplementary Video S1) and in 3 cases this preceded the neuromuscular symptoms. Three‐quarters (6/8) of the patients had febrile seizures, all resolving by the age of 6 years, and none went on to develop epilepsy. There was variable, mild ID (3/8). Cerebellar signs were variably present: nystagmus (3/8; Supplementary Video S2) and ataxia (3/8). Individual 5:I was the only patient with developmental delay and dysmorphism.

**Table 1:** Clinical Features of PIGG Families

Family	1		2	3	4		5	6
Individual	I	II	I	I	I	II	I	I
Variant 1	p.(Trp505*)		p.(Trp678*)	p.(Trp505*)	p.(Trp505*)		c.2735+2T>C	p.Asp876ArgfsTer111
Variant 2	Homozygous		p.(Gly41*)	Homozygous	p.(Val339Gly) p.(Gly19Glu)		p.(Gly278Arg)	Homozygous
Neuropathy phenotype	dHMN	dHMN	dHMN	dHMN	dHMN	dHMN	HMN	dHMN
Motor CB or TD	No	CB/TD	CB/TD	CB	TD and CB/TD	CB and TD	TD	CB and CB/TD
Ethnicity	White British	White British	Irish	Irish	Dutch	Dutch	Cyprus	White Serbian
Sex	M	F	F	F	F	M	F	F
Age at NM symptom (tremor) onset, yr	11 (3)	Teens (9)	Early teens (15)	3 (6)	Early childhood (4)	12 (4)	4 mo (?)	2 (9)
Age at assessment (current), yr	48 (57)	43 (52)	19 (27)	19 (19)	22 (23)	20 (21)	12 (27)	43 (43)
Presenting NM symptom	Sprained ankles	Tripping	Tripping/falls	Tripping	Leg pain during exercise	Spontaneous movements in calves	Hypotonia	Frequent falls
CMTES (age, yr)	5 (53)	4 (47)	5 (19)	3 (19)	NA	NA	NA	7 (43)
Lower limb weakness (MRC grade)	Distal (4+/5)	Distal (4+/5)	Distal (4/5)	Distal (4+/5)	Distal (4+/5)	Distal (4/5)	Distal (4+/5) proximal (4/5)	Distal (3/5)
Upper limb weakness	Nil	Nil	Nil	Nil	Nil	Nil	Distal (4/5)	Distal (4/5)
Nystagmus	No	Gaze‐evoked torsional	Gaze‐evoked	No	No	No	Gaze evoked	No
Strabismus	No	Yes	No	No	No	No	Yes	No
Ataxia	No	No	Finger nose ataxia	Mild heel shin ataxia	No	No	Mild gait ataxia	No
Postural tremor	Yes	Yes	Yes	Yes	Yes	Yes	Yes	Yes
ID /cognitive impairment	No	No	Mild ID	No	Learning problems	No	Mild ID	No
Childhood febrile seizures (until, yr)	No	Yes, 1 episode	Yes (4)	Yes (4)	Yes (6)	Yes (4)	Yes (18 mo)	No
Cerebellar atrophy	Mild superior vermis	No	Yes	No	No	Unknown	No	Unknown
Nerve thickening (modality)	Yes (MRI thigh)	Yes (MRI spine)	Yes (MRI spine)	Yes (MRI spine)	Yes (Nerve US)	No	No	Unknown

CB = conduction block; CMTES = Charcot–Marie‐Tooth Examination Score; CS = conduction slowing; (d)HMN = (distal) hereditary motor neuropathy; F = female; ID = intellectual disability; LL = lower limb; M = male; mo = months; MRC = medical research council; MRI = magnetic resonance imaging; NA = not applicable; NM = neuromuscular; TD = temporal dispersion; US = ultrasound.

*Note*: CD/TD signifies significant amplitude decrease but limitations of the study preclude delineation between CB versus TD.

**Figure 1:**
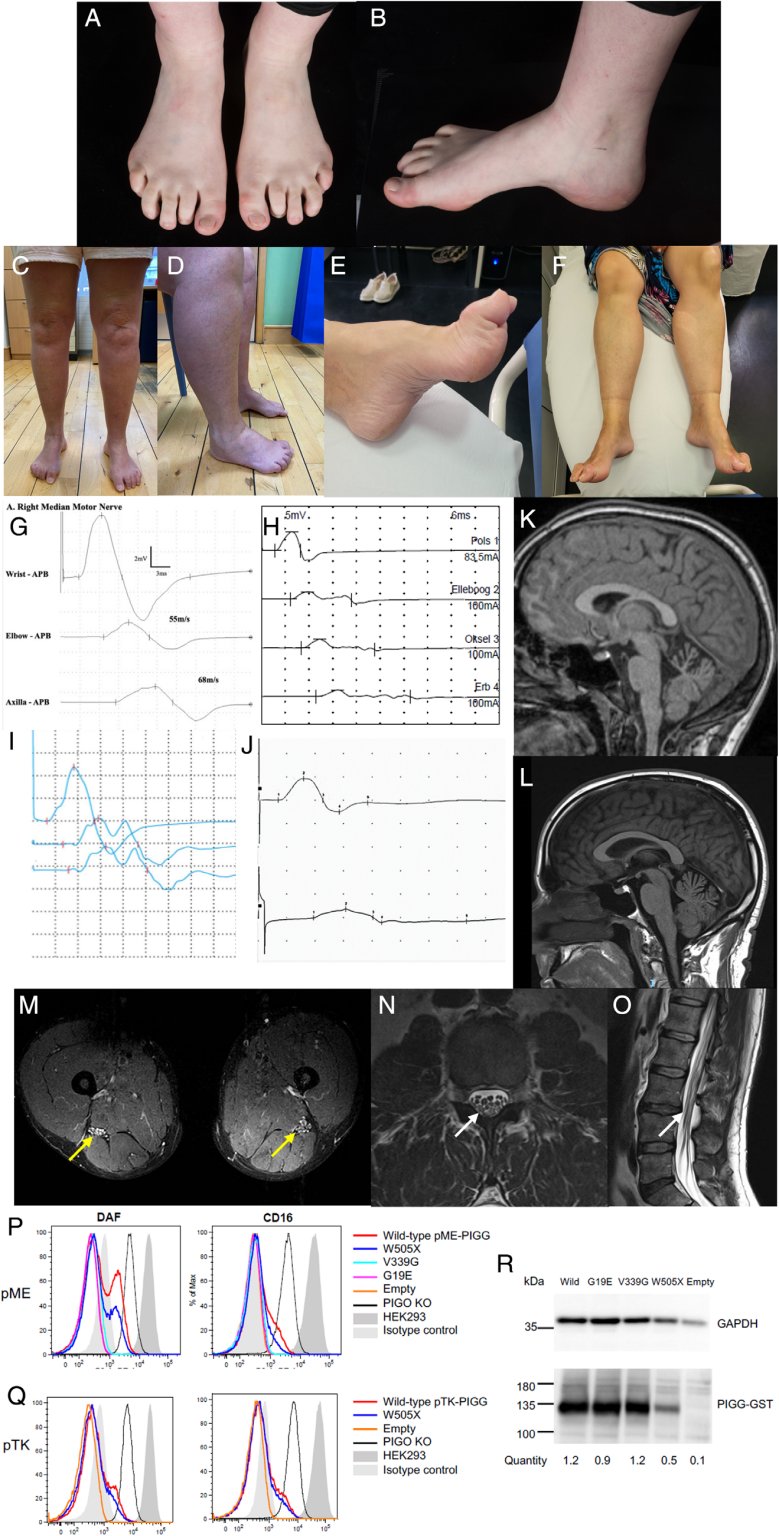
(A–F) Photography of lower limbs showing foot deformity and distal wasting (A and B patient 2:I, C and D patient 1:II, E and F patient 6:I); (G–J) Waveforms of motor nerve conduction studies showing conduction block and temporal dispersion (G patient 2:1 right median, H patient 4:I right median, I patient 5:I right ulnar, J patient 6:I left median); (K–L) MRI of the brain, sagittal T1 views showing prominent (K patient 2:I) and mild (L patient 1:I) superior vermian atrophy; (M) MRI of the thigh of patient 1:I (T2 TIRM axial view) shows thickening of sciatic nerve bilaterally returning high signal (*yellow arrows*); (N, O) MRI of the lumbar spine of patient 1:I shows thickened intradural roots within the cauda equina (N axial T2, O sagittal T2, *white arrows*); (P–R) PIGO/PIGG DKO HEK293 cells (permanently expressing CD16) were transfected with the strong promoter (pME, P) or weaker promoter (pTK, Q) driven wild‐type or mutant PIGG. Two days later, fluorescence‐activated cell sorting (FACS) analysis (P, Q) and Western Blotting (R) were performed. FACS analysis – X‐axis is relative cell number, Y‐axis is fluorescent intensity of phycoerythrin. Western blots – quantity was calculated by the band intensities of PIGG‐GST normalized with the band intensities of GAPDH (loading control) and with luciferase activity (transfection control). MRI = magnetic resonance imaging. [Color figure can be viewed at www.annalsofneurology.org]

The neuropathy was generally a mild, minimally progressive, distal, motor neuropathy, with only 4 of 8 patients having minor sensory symptoms or signs. Six of 8 patients had a foot deformity (Fig1A–F) and subtle upper motor neuron signs were seen in 3 of 8 patients. Individual 4:II showed striking spontaneous muscle activity after exercise, but not at rest, although further characterization with electromyography (EMG) was not possible (Supplementary Video S3).

Neurophysiology (Table 2) showed a motor neuropathy with normal sensory conduction in all individuals. The studies of 7 of 8 patients demonstrated variable motor conduction block (CB), temporal dispersion, and sometimes a combination of the two (Fig1G–J and Supplementary Fig S1), mostly in the forearm in the median and ulnar nerves. This is assessed by reduced motor amplitudes to proximal stimulation with either a fall in motor response area (motor CB) or prolongation of motor response (dispersion) or both. There was also some minor motor slowing seen generally but not within the demyelinating range. The only convincing conduction slowing was the outlying median velocity of 36.3 m/s in the forearm of individual 6:I. Where performed, imaging confirmed cerebellar atrophy in 2 of 6 individuals (Fig1K, L) and nerve thickening in 5 of 7 individuals (magnetic resonance imaging [MRI] or nerve ultrasound; Fig1M–O and Supplementary S1). Individual 5:I spontaneously improved with time, which was confirmed by serial neurophysiological studies (Supplementary Table S2).

**Table 2:** Upper Limb Motor Nerve Conduction Studies

Family	1		2	3	4		5	6
Individual	I	II	I	I	I	II	I	I
Age at study	48	43	19	12	21	19	24	43
Summary	LD motor neuropathy	LD motor neuropathy with median CB/TD	LD motor neuropathy with median and ulnar mixed CB/TD	LD motor neuropathy with ulnar CB	Motor neuropathy with median TD and mixed CB/TD in ulnar	Motor neuropathy with ulnar CB and median TD	LD motor neuropathy with ulnar TD	LD motor neuropathy with intermediate velocity in median and median CB and ulnar CB/TD
**Median nerve**								
Median CMAP (wrist) mV	4.2	7.4	6.1	5.8	8.8	8.0	5.5	4.6
Median CMAP (elbow) mV	3.3	3.8	1.4	4.1	3.4	5.1	3.6	1.9
Median DML ms	4.7	3.9	3.1	3.5	3.4	4.8	3.5	3.5
Median NCV (wrist‐elbow) m/s	48	47	53	54	49	52	50	36.3
Median F latency (ms)	35	30	36	ND	32	34	33.4	ND
**Ulnar nerve**								
Ulnar CMAP (wrist) mV	5.9	5.8	4.1	4.3	5.7	6.2	4.8	2.7
Ulnar CMAP (below elbow) mV	3.7	3.9	0.5	2.6	2.9	3.2	2.3	1.1
Ulnar CMAP (above elbow) mV	3.1	3.6	0.9	2.6	2.8	3.2	2.1	ND
Ulnar DML ms	3.7	4.4	2.9	3.3	3	2.8	3.1	3.75
Ulnar NCV (wrist‐below elbow) m/s	55	53	67	42	49	61	53	47.3
Ulnar NCV (around elbow) m/s	50	56	67	110	57	44	85	ND
Ulnar F latency ms	52	30	40	ND	29	43	ND	ND

*Note*: All nerves studied on the right except patients 1:I, median nerve of 5:I and 6:I.

CB = conduction block; CMAP = compound motor action potential; DML = distal motor latency; LD = length‐dependent; NCV = nerve conduction velocity; ND = not done; TD = temporal dispersion; UL = upper limb.

### Genetics

Six variants in *PIGG* were detected (see Table 1, variant classification Supplementary Table S3) including the previously reported c.1515G>A p.(Trp505*) (pathogenic),^3^, ^8^ c.832G>A p.(Gly278Arg) (variant of uncertain significance [VUS]),^3^, ^9^ and the novel c.121G>T p.(Gly41*) (pathogenic), c.2034G>A p.(Trp678*) (pathogenic), c.2735+2T>C (likely pathogenic), c.2625dup p.(Asp876ArgfsTer111) (likely pathogenic), and the pair of variants in *cis* c.56G>A p.(Gly19Glu) and c.1016T>G p.(Val339Gly) (both likely pathogenic). No other relevant variants were detected in any individuals; variants unrelated to the disease detected in individuals 2:I and 3:I are detailed in Supplementary Table S3.

### Functional Studies and Emm Blood Group Testing

Three variants were tested in the *PIGO/PIGG* DKO system. Restoration of the GPI‐AP expression on *PIGO/PIGG* DKO cells by transfection with WT or variant *PIGG* cDNA was compared (Fig1P strong promoter, and Fig1Q weak promoter), and *PIGG* variants’ expression analyzed by Western blotting (Fig1R). As previously shown, transfection of WT *PIGG* shows decreased activity when C‐terminally tagged compared with non‐tagged *PIGG* in the *PIGO* KO^3^ (red compared with black lines, Fig1P, Q). However, Val339Gly and Gly19Glu variants had null enzymatic activity even if they were transfected with the strong promoter driven construct (Fig1P turquoise and magenta lines), but they expressed protein levels similar to WT *PIGG* (Fig1R). The nonsense Trp505* variant showed decreased activity compared to WT *PIGG* driven by either the weak or strong promoter (Fig1P, Q blue compared with red lines) but retained residual activity because of partial expression of full‐length protein (Fig1R).

In the 2 members of family 4 (both compound heterozygous for Trp505* in *trans* with Val339Gly and Gly19Glu), there was no Emm antigen expression detectable on the red cells of the patients, confirming Emm negative blood group status (Supplementary Table S4).

## Discussion

We report the first series of patients with biallelic variants in *PIGG* and a motor neuropathy associated with prominent tremor, febrile seizures, and variable cerebellar involvement (present in 3/8 patients, comparable to a previous study) but without ID in the majority.^3^ Prior studies have reported hypotonia and diminished deep tendon reflexes (DTRs), suggesting a motor neuropathy, but without confirmatory neurophysiology.^2^, ^3^ The neuropathy, although relatively mild, is the unifying feature in this cohort. The prominence of early‐onset postural tremor seen in 4 cases without cerebellar signs, favors a neurogenic origin, supported by all cases of tremor reported by Tremblay‐Laganière et al (5/21) having either diminished DTRs or hypotonia.^3^ A notable feature of the neuropathy is the prominent motor CB and temporal dispersion, with no major slowing of motor conduction in the segments with CB/dispersion. The presence of these features at non‐compression sites typically suggests an inflammatory etiology although CB/dispersion is a feature of some inherited neuropathies.^10^ More unusual is motor CB or dispersion occurring in a motor predominant, inherited neuropathy, only reported rarely (*SORD*, *PLEKHG5*, and *SIGMAR1*).^11^, ^12^, ^13^ The improvement of individual 5:I over time, for which the mechanism is not understood, again contributes to *PIGG*‐neuropathy acting as an inflammatory mimic.

The allele frequency of Trp505* in population databases (1719/1614208 alleles, heterozygous frequency 1.06 × 10^−3^, plus 2 homozygotes, in GnomADv4.0.0) in the context of a rare disease, merits discussion. Although this is compatible with a rare recessive disorder, the corresponding disease prevalence is expected to be higher.^14^ An explanation for this discordance is the mild and variable phenotypes previously reported in individuals homozygous for this variant; isolated febrile seizures^3^ and autism.^8^ Similarly, the patients in our cohort (individuals 1:I, 1:II, and 3:I) with homozygous Trp505* have a relatively mild phenotype without the classical features of IGD. This is corroborated by the residual PIGG enzyme activity demonstrated in *PIGO/PIGG* DKO HEK293 cells transfected with the Trp505* mutant; we have confirmed the previously hypothesized residual protein product via likely read through of the nonsense codon.^3^, ^15^ Considering the population frequency and the above evidence, it is therefore likely that Trp505* is a hypomorphic allele, as seen in other IGD disorders,^16^ and homozygotes may have minimal symptoms or do not manifest. Contrastingly both the Val339Gly and Gly19Glu variants (seen in *cis* in individuals 4:I and 4:II) show no enzyme activity and have population allele frequencies in the order of 0.5 to 1  x 10^−5^ in keeping with a complete loss‐of‐function allele.

The role of GPI and GPI‐APs in neurogenesis is clearly evidenced by the characteristic neurological features of IGD caused by almost 20 of the PIG and associated PGAP (post‐GPI attachment to proteins) families of genes.^1^ The function of GPI as an anchor for dozens of cell surface proteins has led to work investigating the effect of the disease causing PIG genes on specific proteins, with disease mechanisms hypothesized.^17^, ^18^, ^19^, ^20^ However, to our knowledge, the exact disease mechanisms of the IGDs has not been elucidated.

All but one of the genes producing an IGD syndrome report associated hypotonia, but apart from 4 cases with variants in *PIGB* described with both axonal and demyelinating polyneuropathies (without accompanying neurophysiological data)^19^ a neuropathy has not been explicitly identified in IGD.

Two broad hypotheses can be considered regarding the pathogenesis of *PIGG*‐related neuropathy and neuropathy in the broader context of this family of genes. First, that GPI is fundamental to peripheral neurogenesis and all IGD syndromes contain a neuropathy as part of their presentation due to aberrant GPI, and the reason this had not been previously demonstrated is that in many of the patients with IGD and severe central nervous system abnormalities or early death, this has not been investigated. Evidence as to which GPI‐AP, or combination of GPI‐APs, is implicated in disease is lacking, but the role of vitamin B6 has previously been postulated; particularly in view of the reported pyridoxine‐responsive seizures in some patients with IGD due to variants in *PIGO* and *PIGS*.^20^, ^21^ Murakami et al noted that the GPI‐AP alkaline phosphate converts the active form of vitamin B6 (pyridoxal 5’‐phosphate [PLP]) to pyridoxal allowing transport across the blood–brain‐barrier. Analogies can be drawn with both the recessive mutations in *PDXK*, which cause a B6‐responsive inherited motor and sensory neuropathy due to reduced enzymatic conversion of pyridoxal to PLP^22^, and the neuropathy caused by nutritional B6 deficiency.^23^ Given the theoretical therapeutic implications, further work to confirm or refute the involvement of alkaline phosphate, and therefore B6, in the neuropathy of patients with IGD, is critical given the reported peripheral neuropathy caused by excess pyridoxine consumption.^23^

Alternatively, the role of glial‐cell‐line‐derived neural growth factor (GDNF) as a survival factor for central and peripheral neurons could be considered for IGD‐related neuropathy. The cellular responses to GDNF have been shown to require the cell surface receptor GDNFR‐α which is a GPI‐AP.^24^ Extrapolating, reduced surface expression of GDNFR‐α due to a PIG gene defect could render GDNF ineffective and result in a peripheral (and central) neuropathy.

The component of GPI that binds to the cell membrane is the phospholipid phosphatidylinositol. Independent of GPI, this moiety exists in 7 phosphorylation states, termed phosphoinositides, each controlling numerous cellular processes. Defects in these phosphorylation pathways are implicated in numerous neurological disorders, many of which phenotypically overlap with IGD syndromes.^25^ Mutations in some phosphoinositide metabolism genes (*FIG4*, *PTEN*, *MTMR2*, and *SBF1*) also cause neuropathies with conduction slowing and/or CB,^26^, ^27^, ^28^ suggesting a more fundamental role of GPI in the pathophysiology of this inherited neuropathy, independent of the GPI‐APs that are affected.

The above arguments, however, do not account for the specific phenotype seen in *PIGG*‐related neuropathy. The second hypothesis is that defects in *PIGG* have specific pathological effects on peripheral nerve causing this unusual neuropathy phenotype. Work by Ishida et al has furthered understanding of the role of PIGG in cell‐surface protein anchoring. They challenged the established notion that GPI‐APs are bound to the third mannose of GPI. Through a series of experiments, they showed that the EtN‐P on the second mannose, previously thought to be discarded after the GPI‐AP was anchored, was retained and itself formed the bridge to some selected GPI‐APs.^7^ Comparing the expression levels of various GPI‐APs in *PIGG* KO HEK293 cells with those rescued by transient co‐transfection with *PIGG* cDNA, a number of GPI‐APs were PIGG‐dependent. The largest dependence was seen in NTNG2 (recessive variants in *NTNG2* also cause a neurodegenerative disorder),^29^ but importantly CNTN1 was among the most PIGG‐dependent. This is particularly relevant given the known acquired autoimmune paranodopathy caused by antibodies against CNTN1.^30^ The paranodopathy causes an “acquired” neurophysiological picture with conduction slowing, dispersion and CB, but histopathology had demonstrated that the pathology (and the resultant neurophysiological features) lies in disruption of structures at the Node of Ranvier, and not in the myelin.^31^ The neurophysiological findings in our *PIGG*‐neuropathy cohort, I demonstrating CB and dispersion in an otherwise predominantly axonal neuropathy, could theoretically place the pathology at the node/paranode, and in conjunction with evidence that CNTN1 is PIGG‐dependent, allow speculation that variants in *PIGG* cause a genetic paranodopathy. Although nonspecific, the consistency of the early onset neuropathic tremor in out cohort mirrors the frequent tremor seen in CNTN1‐related paranodopathy.^32^ Last, CD59 is another GPI‐AP and biallelic variants in *CD59* cause an inherited immune‐mediated neuropathy. Although not the most PIGG‐dependent GPI‐AP,^7^ parallels can be drawn between this condition and the neurophysiological features in our cohort, particularly the spontaneous improvement of individual 5:I.^33^

The importance of GPI in human health and disease, is illustrated by the fact that in addition to the neurological phenotype, *PIGG* has been demonstrated to define the rare blood group system Emm.^5^ Duval et al have shown that the Emm antigen is not, like other blood groups, a GPI‐AP but free, unlinked, GPI.^4^ The epitope for Emm was shown to be the EtN‐P on the second and third mannose groups; PIGG facilitates the addition of the second mannose and individuals with biallelic *PIGG* variants have shown absent Emm expression on RBC, that is, Emm negativity.^4^, ^5^, ^34^ Patients with IGD and variants in *PIGO* (the gene facilitating the addition of EtN‐P to the third mannose) showed decreased but not absent Emm expression on RBCs, whereas those with *PIGN* and *PIGA* variants had normal of upregulated Emm levels.^4^ In our study, the negative Emm blood group status of the siblings of family 4 support the literature that *PIGG* causes both neurological and hematological manifestations, and acts as further evidence for the pathogenicity of Trp505* in *trans* with Val339Gly and Gly19Glu.

In conclusion, our series of patients with biallelic variants in *PIGG* broadens the phenotype of *PIGG*‐related disease to include a motor‐predominant neuropathy associated with tremor, motor CB, and dispersion, that can neurophysiologically mimic acquired disease, without major ID. *PIGG* should be considered in all unsolved cases of inherited motor‐predominant neuropathy. Further clinical studies, including neurophysiology, are needed to characterize the hypotonia seen in other PIG‐related disorders to delineate the presence, or absence, of neuropathy, and the neurophysiological phenotype. More work is needed to further understand the mechanism of *PIGG*‐related neuropathy. Application of existing methods to demonstrate reduced PIGG activity or expression for our unstudied novel variants would add support to their pathogenicity, but are unlikely to aid mechanistic understanding. Ideally, studies would investigate nerve histopathology of affected individuals, tissue‐specific gene expression of GPI‐APs, and immunological reactivity to anti‐CNTN1 (and other) antibodies of the paranode of PIGG deficient neurons, or other cell lines, challenging the hypothesis of a genetic paranodopathy. Alternatively, disease models in patient‐derived induced pluripotent stem cell‐derived motor neurons, or animal models, could shed light on pathological mechanisms.

## Author Contributions

C.J.R., A.O'C., P.M.C., S.M., and M.M.R. contributed to the conception and design of the study. C.J.R., A.O'C., N.E.V., W.vR., E.Z.P., S.P., P.C.L., M.S., E.vB., V.I., B.H., J.C.M., J.B., Y.M., M.L., S.M.M., and M.M.R. contributed to the acquisition and analysis of data. C.J.R., A.O'C., Y.M., and M.M.R. contributed to drafting the text or preparing the figures.

## Potential Conflicts of Interest

Nothing to report.

## Supporting information


**Video S1.** Tremor in individual 1:II.

**Video S2.** Nystagmus in individual 1:II.

**Video S3.** Spontaneous muscle activity after exercise in individual 4:II.

**Figure S1.** Further examples of conduction block and temporal dispersion, MRI images showing nerve root thickening, nerve ultrasound demonstrating patchy nerve thickening.

**Figure S2.** Uncropped Western Blots.

**Table S1.** Additional clinical features.

**Table S2.** Serial nerve conduction studies in individual 5:I.

**Table S3.** Variant classification.

**Table S4.** Hemagglutination results EMM antigen typing.

## Data Availability

The data that support the findings of this study are available from the corresponding author upon reasonable request.
