# Supplementary material for: Recessive Variants in PIGG Cause a Motor Neuropathy with Variable Conduction Block, Childhood Tremor, and Febrile Seizures: Expanding the Phenotype
Source: Ann Neurol. 2024 Oct 23;97(2):388–96. doi: 10.1002/ana.27113 (PMC11740278; doi:10.1002/ana.27113)

| **Supplementary Figure 1** – Additional neurophysiology data showing waveforms of upper limb motor nerves, MR imaging demonstrating thickened intradural nerve roots and nerve ultrasound demonstrating patchy nerve thickening | |
| --- | --- |
| Patient 2:1 Right ulnar | Patient 4:1 Right ulnar |
| 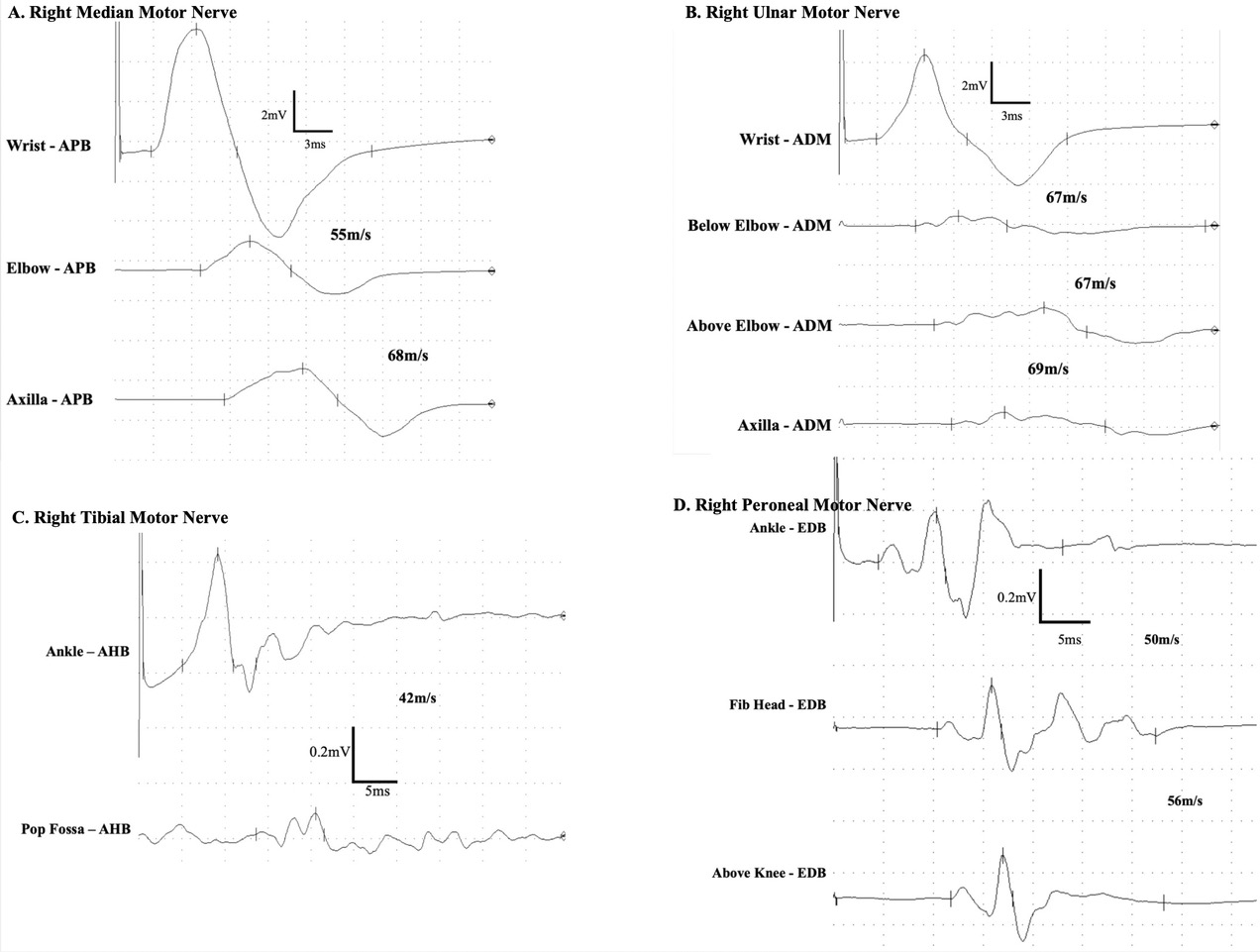 | 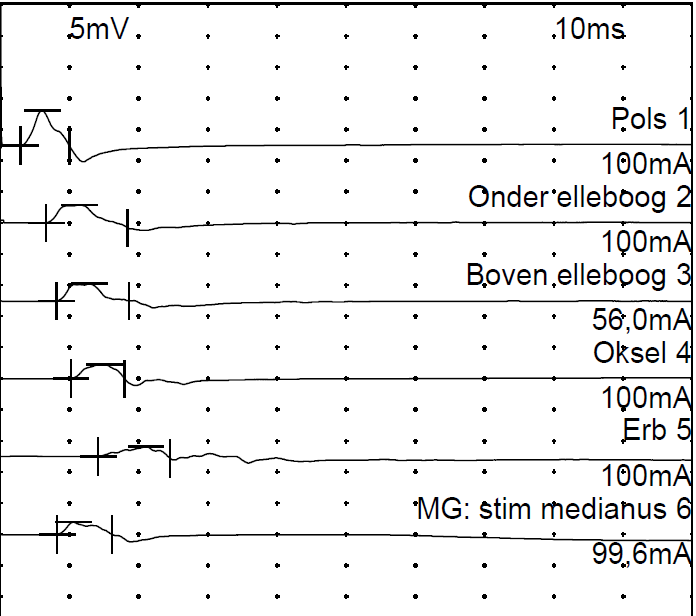 |
| Patient 6:1 Left Ulnar |  |
| 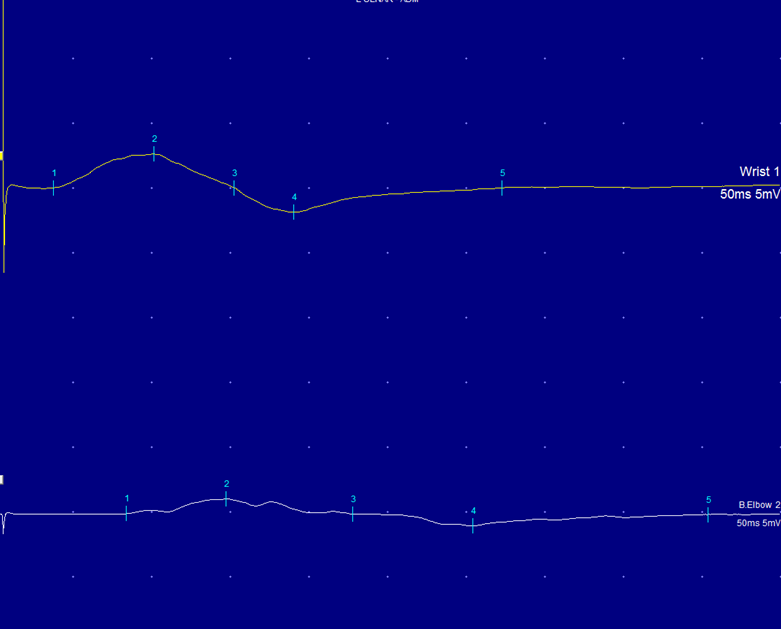 |  |
| Patient 1:II MRI Lumbar Spine; Axial T2 | Patient 1:II MRI Lumbar Spine; Sagittal T2 |
| 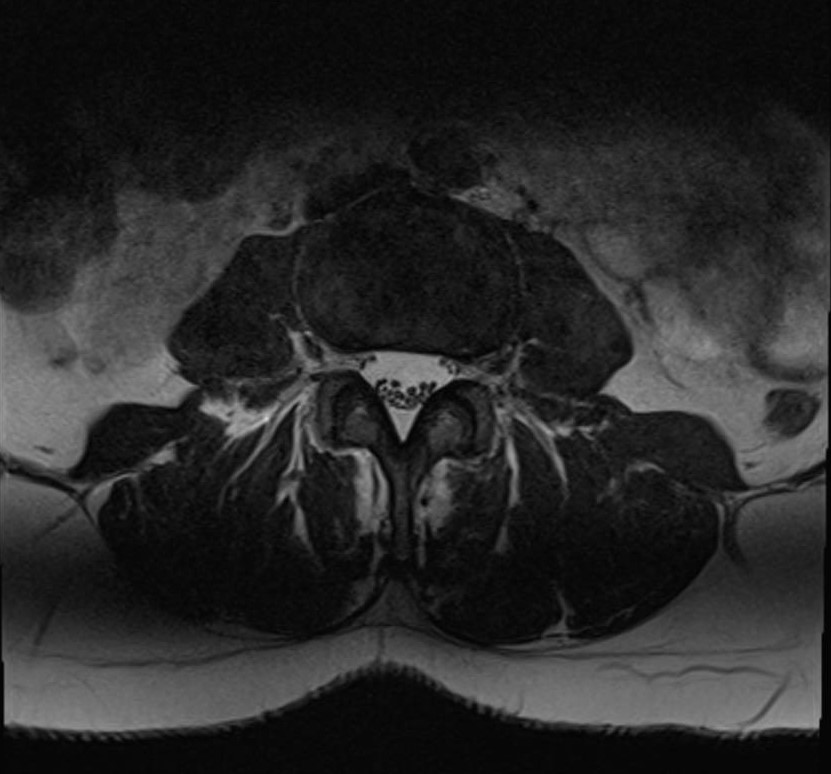 | 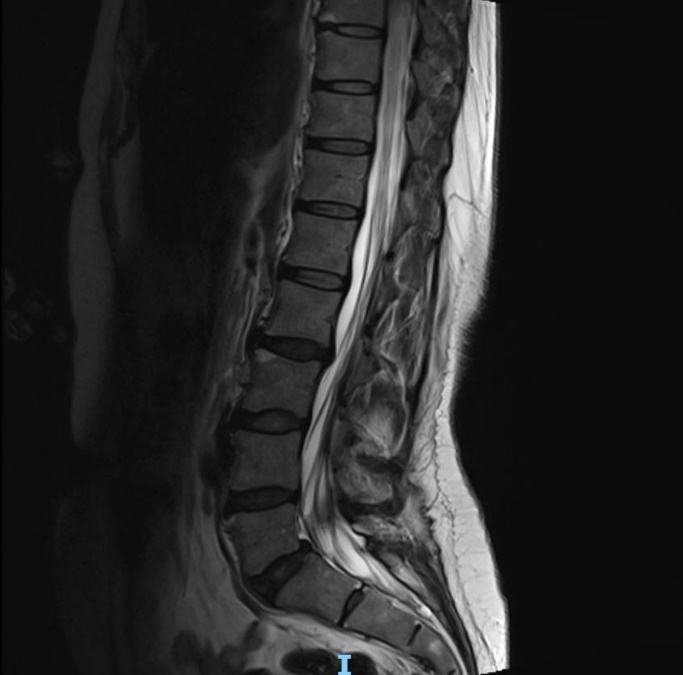 |

| Patient 2:I MRI Lumbar Spine; Axial T2 | Patient 2:I MRI Lumbar Spine; Sagittal T2 |
| --- | --- |
| 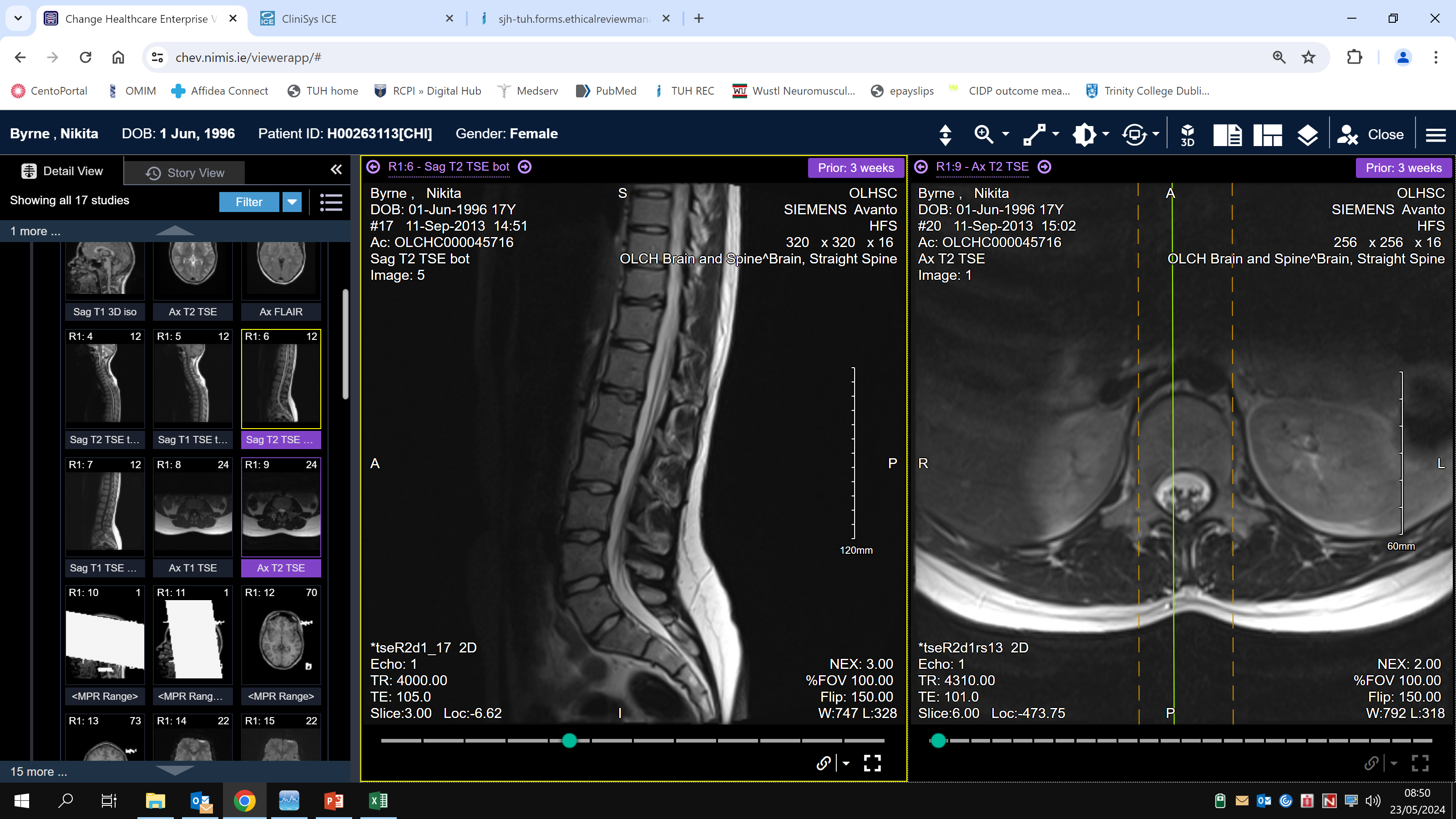 | 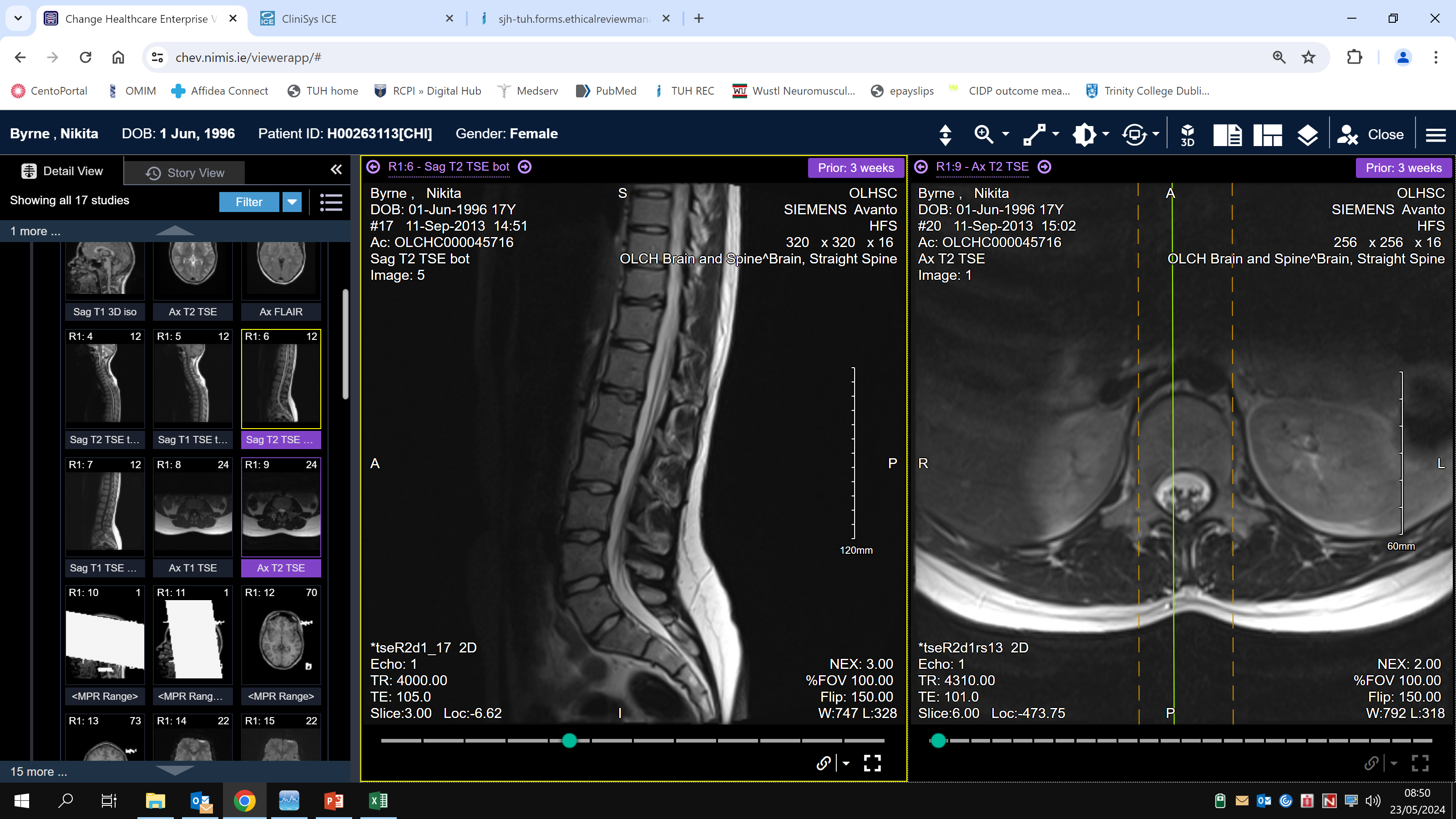 |
|  |  |
|  |  |
|  |  |
|  |  |
|  |  |
|  |  |
|  |  |
|  |  |
|  |  |
|  |  |
| Patient 3:I MRI Lumbar Spine; Axial T2 | Patient 3:I MRI Lumbar Spine; Sagittal T2 |
| 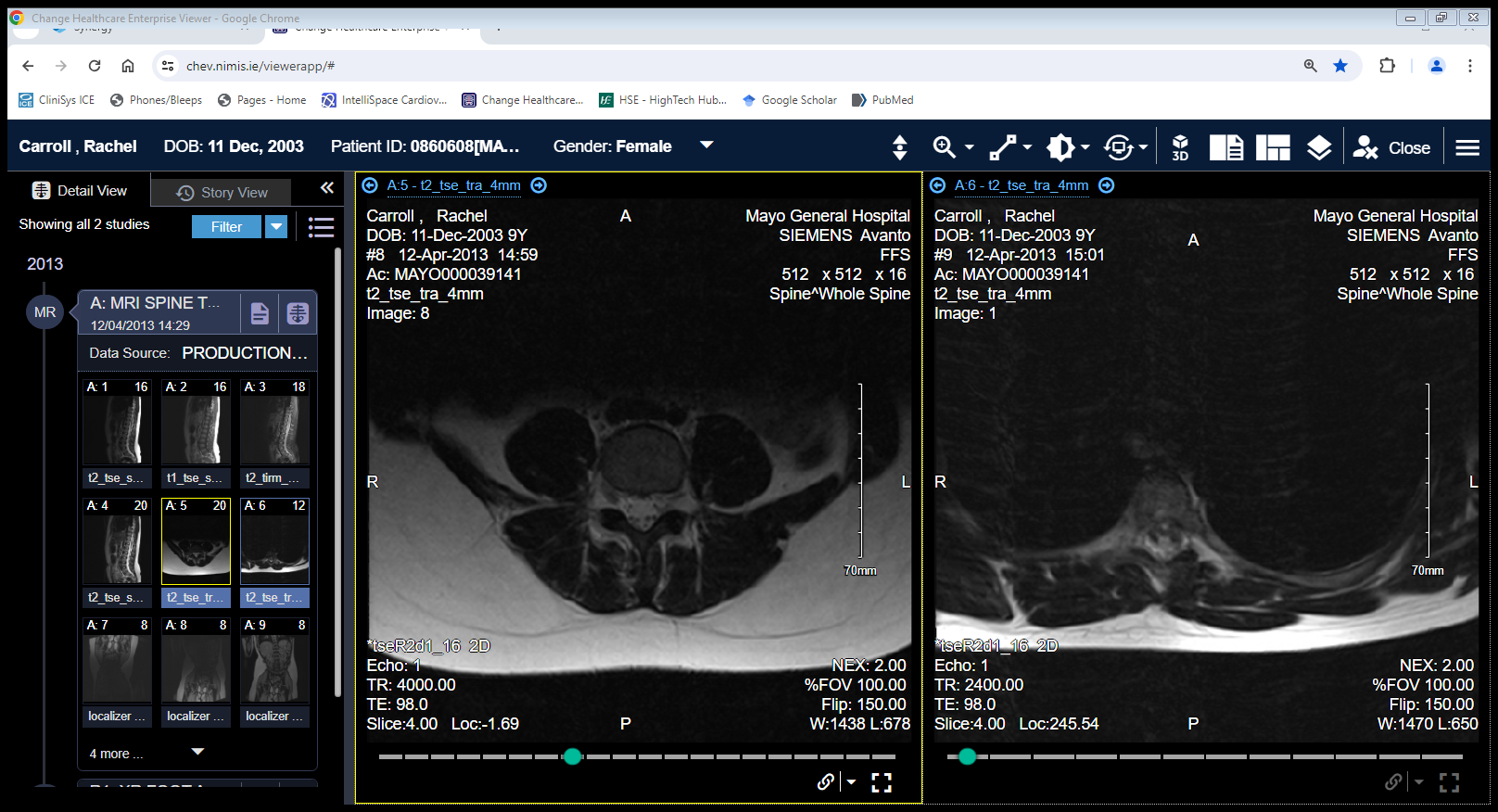 | 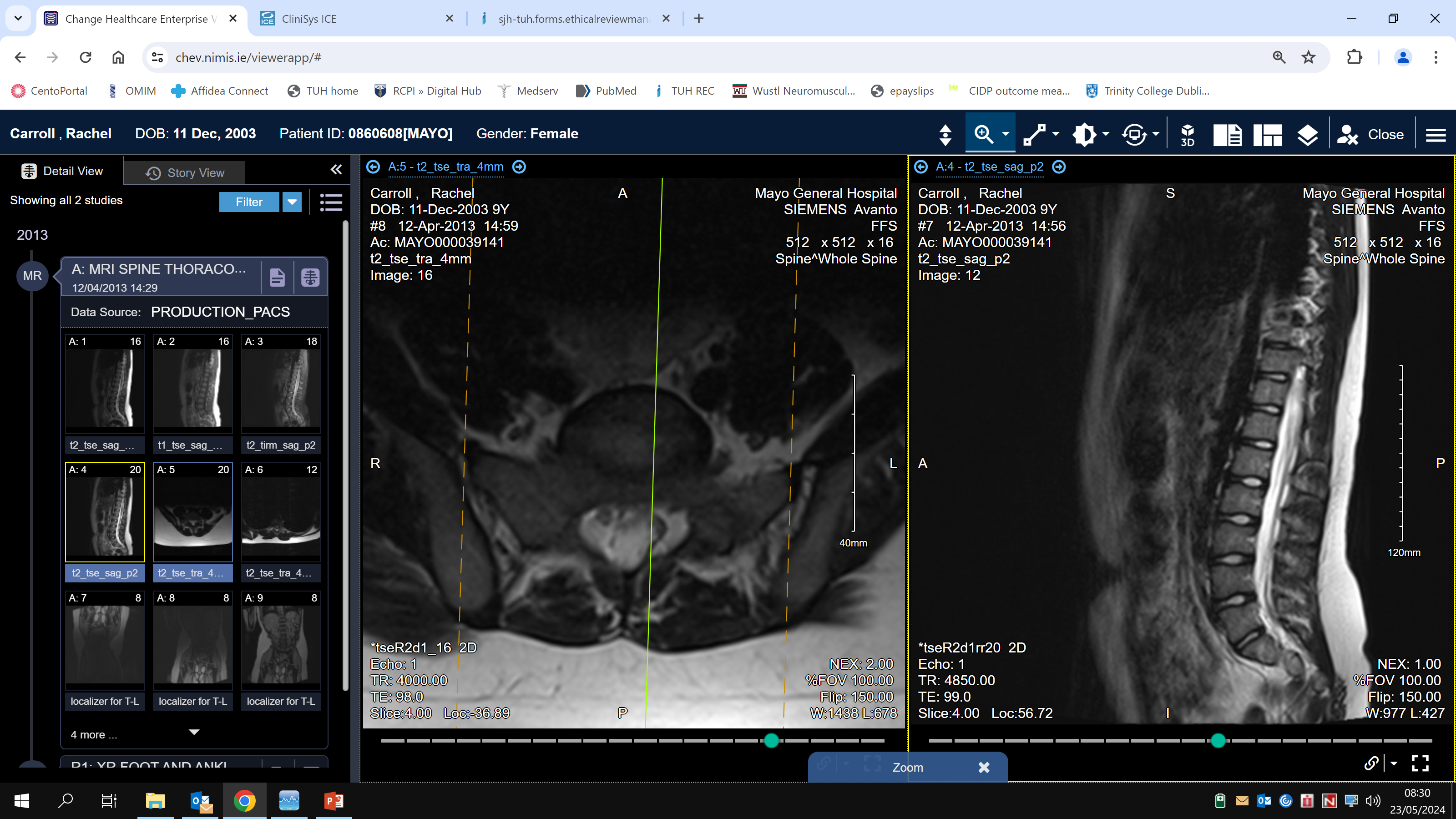 |
| Yellow arrows indicate mild-moderately thickened intradural nerve roots within the cauda equina | |


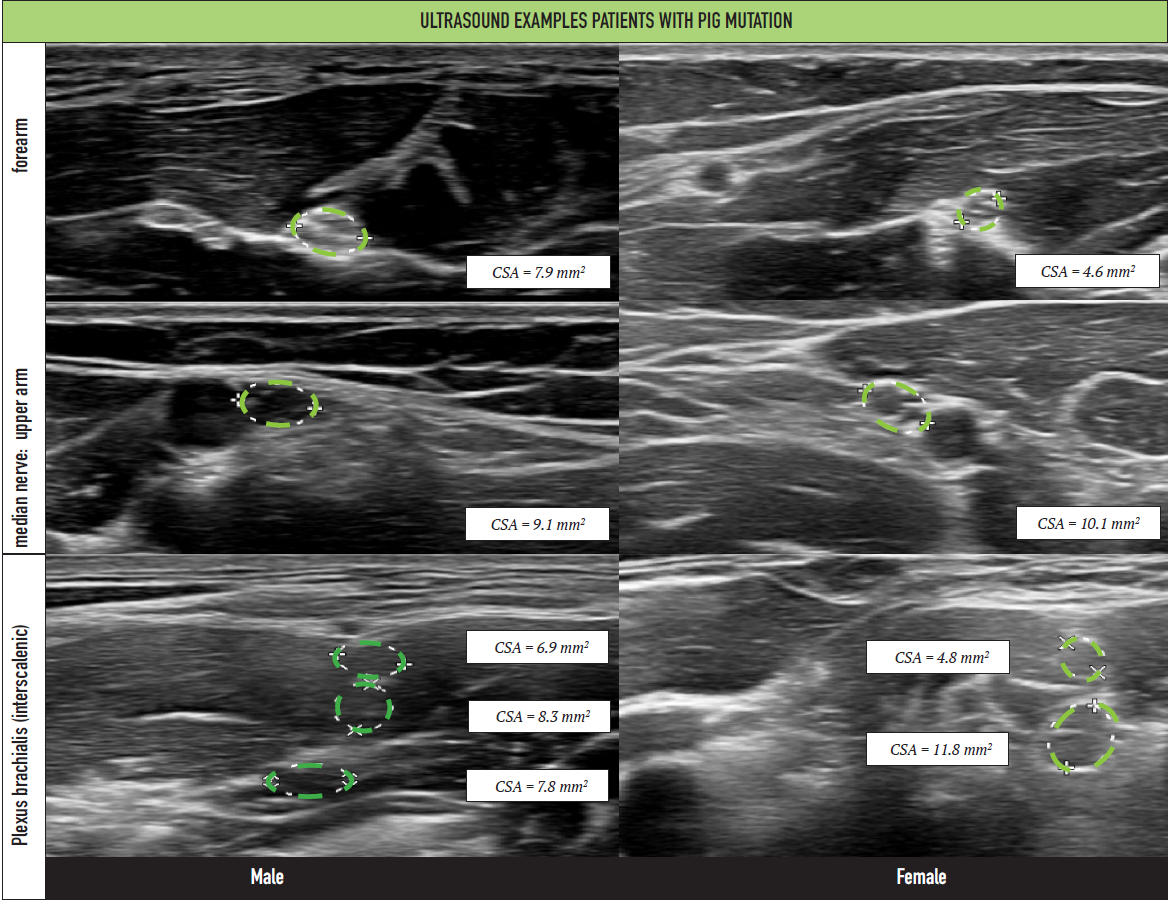
Nerve ultrasound of male (Patient 4:II) and female (Patient 4:I). Normal values for median nerve in forearm <9mm^2^, median nerve in upper arm <10mm^2^, and brachial plexus <9mm^2^. Nerve enlargement seen in female median nerve in upper arm, and middle trunk.

C6 nerve root of patient 4:I is enlarged (normal <12 mm^2^)


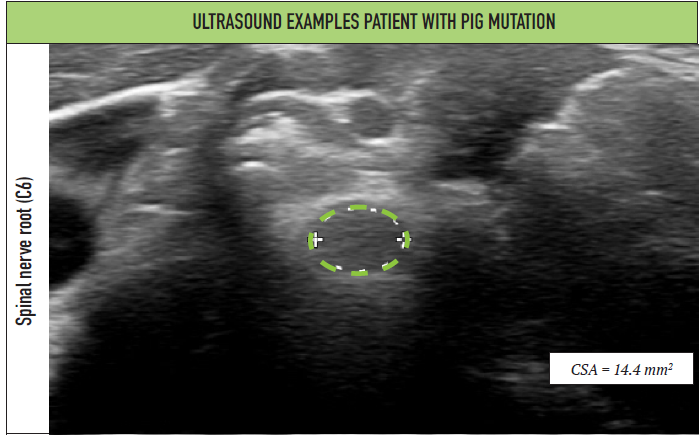

Supplement: Supplementary file 4 — Figure S1. Further examples of conduction block and temporal dispersion, MRI images showing nerve root thickening, nerve ultrasound demonstrating patchy nerve thickening. [file ANA-97-388-s006.docx]
