# Supplementary figures and images for: Recessive Variants in PIGG Cause a Motor Neuropathy with Variable Conduction Block, Childhood Tremor, and Febrile Seizures: Expanding the Phenotype
Source: Ann Neurol. 2024 Oct 23;97(2):388–96. doi: 10.1002/ana.27113 (PMC11740278; doi:10.1002/ana.27113)

**Supplementary Figure 2**

**Uncropped blots**

GADPH

**

**

PIGG-GST


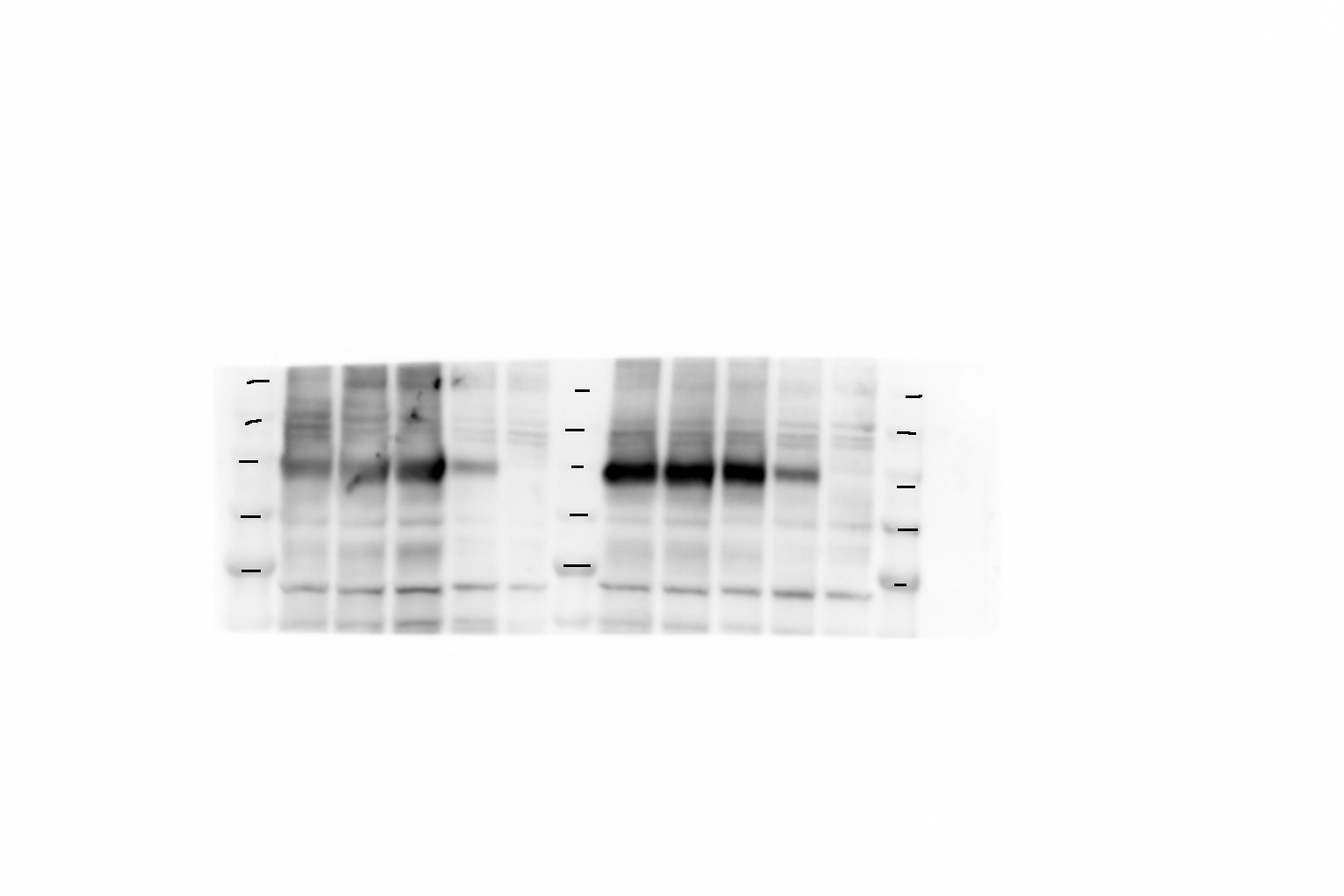

Supplement: Supplementary file 5 — Figure S2. Uncropped Western Blots. [file ANA-97-388-s005.docx]
