# Supplementary material for: Recessive Variants in PIGG Cause a Motor Neuropathy with Variable Conduction Block, Childhood Tremor, and Febrile Seizures: Expanding the Phenotype
Source: Ann Neurol. 2024 Oct 23;97(2):388–96. doi: 10.1002/ana.27113 (PMC11740278; doi:10.1002/ana.27113)
Supplement: Supplementary file 6 — Table S1. Additional clinical features. [file ANA-97-388-s009.docx]

| **Family** | 1 | | 2 | 3 | 4 | | 5 | 6 |
| --- | --- | --- | --- | --- | --- | --- | --- | --- |
| **Individual** | I | II | I | I | I | II | I | I |
| **Variant 1:Nucleotide** | c.1515G>A | | c.2036G>A | c.1515G>A | c.1515G>A | | c.2735+2T>C | c.2625dup |
| **Variant 1:Amino acid** | p.(Trp505*) | | p.(Trp678*) | p.(Trp505*) | p.(Trp505*) | | p.? | p.Asp876ArgfsTer111 |
| **Variant 2:Nucleotide** | Homozygous | | c.121G>T | Homozygous | c.1016T>G c.56G>A | | c.832G>A | Homozygous |
| **Variant 2:Amino acid** | Homozygous | | p.(Gly41*) | Homozygous | p.(Val339Gly) p.(Gly19Glu) | | p.(Gly278Arg) | Homozygous |
| **Sensory symptoms** | No | No | LL paraesthesia | No | No | No | Numbness | No |
| **Sensory signs** | UL: pinprick  Reduced to wrist | UL: Reduced in fingers | LL:  Reduced to ankle | No | No | No | No | No |
| **Reflexes Ankle/knee/UL** | Absent/ normal/  brisk | Absent/ absent/ normal | Normal/ normal/ normal | Normal/ normal/ normal | Reduced/ reduced/ reduced | Reduced/ reduced/ Normal | Normal/ Brisk/ Brisk | Absent/absent/brisk |
| **Babinski** | Negative | Negative | Negative | Negative | Negative | Negative | Negative | Negative |
| **Gait** | Inturning feet | Good; extended toes | Broad based, bilateral foot drop | Narrow base, inturning feet | Minimal difficulties | Subtle foot drop | Mild broad-based gait | Steppage |
| **Foot deformities** | Pes cavus, hammer toes | high arches, clawed toes | Pes cavus, hammer toes | Pes Cavus, hammer toes | No | No | Pes cavus, hammer toes | Pes cavus, hammer toes |
| **Genetic testing** | Hereditary motor neuropathy panel, WGS quad | WGS quad | *PMP22* dosage normal, Bristol Neuropathy panel, WES | Trio WES, Centogene WGS | Trio WES | Gene panel testing (negative),  PIGG testing | Karyotype, FISH 22q11, *FMR1*, Microarray, M-FISH, Trio WES | *PMP22* dosage, WES |
| **Supplementary Table 1 Additional clinical features of PIGG families** LL lower limb; UL upper limb; WES whole exome sequencing; WGS whole genome sequencing | | | | | | | | |
