# Supplementary material for: Recessive Variants in PIGG Cause a Motor Neuropathy with Variable Conduction Block, Childhood Tremor, and Febrile Seizures: Expanding the Phenotype
Source: Ann Neurol. 2024 Oct 23;97(2):388–96. doi: 10.1002/ana.27113 (PMC11740278; doi:10.1002/ana.27113)
Supplement: Supplementary file 7 — Table S2. Serial nerve conduction studies in individual 5:I. [file ANA-97-388-s003.docx]

| **Family number** | 5 | | |
| --- | --- | --- | --- |
| **Individual number** | I | | |
| Age at study | 11 | 18 | 24 |
| MOTOR CONDUCTION |  |  |  |
| **Median nerve** |  |  |  |
| Median CMAP (wrist) mV | 1.1 | 2.5 | 7.8 |
| Median CMAP (elbow) mV | 0.8 | 1.7 | 5.0 |
| Median DML ms | 3.8 | 3.6 | 3.64 |
| Median NCV (wrist-elbow) m/s | 57.9 | 55.0 | 56.9 |
| Median F latency (ms) | ND | 24.1 | 33.4 |
| **Ulnar nerve** |  |  |  |
| Ulnar CMAP (wrist) mV | 1.8 | 3.0 | 4.8 |
| Ulnar CMAP (below elbow) mV | 1.4 | 1.5 | 2.3 |
| Ulnar CMAP (above elbow) mV | 1.2 | 1.5 | 2.1 |
| Ulnar DML ms | 2.2 | 4.0 | 3.1 |
| Ulnar NCV (wrist-below elbow) m/s | 46.7 | 47.5 | 53.4 |
| Ulnar NCV (around elbow) m/s | 60.5 | 53.1 | 84.7 |
| Ulnar F latency ms | 26.1 | absent | ND |
| **Lower limb** | | | |
| Tibial CMAP (ankle – AH) mV | Absent | Absent | 0.24 |
| Tibial DML ms | absent | Absent | 11.2 |
| Peroneal CMAP (ankle – EDB) mV | 0.1 | 0.1 | 1.44 |
| Peroneal DML ms | 7.5 | 6.6 | 7.0 |
| Comment | All right side | All right side | All right side |

**Supplementary Table 2**

**Serial nerve conduction studies in individual 5:I** performed by the same operator, showing improvement with time that corresponded with clinical improvement. Aged 8 years there was intrinsic hand muscle weakness, which has improved to normal power by age 21. Cerebrospinal fluid (CSF) analysis performed in the first decade was normal
