# Supplementary material for: Recessive Variants in PIGG Cause a Motor Neuropathy with Variable Conduction Block, Childhood Tremor, and Febrile Seizures: Expanding the Phenotype
Source: Ann Neurol. 2024 Oct 23;97(2):388–96. doi: 10.1002/ana.27113 (PMC11740278; doi:10.1002/ana.27113)
Supplement: Supplementary file 8 — Table S3. Variant classification. [file ANA-97-388-s008.docx]

| **Variant nucleotide** | **c.56G>A** | **c.121G>T** | **c.832G>A** | **c.1016T>G** | **c.1515G>A** | **c.2034G>A** | **c.2625dup** | **c.2735+2T>C** |
| --- | --- | --- | --- | --- | --- | --- | --- | --- |
| **Variant amino acid** | **p.(Gly19Glu)** | **p.(Gly41*)** | **p.(Gly278Arg)** | **p.(Val339Gly)** | **p.(Trp505*)** | **p.(Trp678*)** | **p.Asp876ArgfsTer111** | **p.?** |
| Hg19 | 4-493180-G-A | 4-493245-G-T | 4-502690-G-A | 4-509876-T-G | 4-515631-G-A | 4-517667-G-A | 4-527659-T-TA | 4-527772-T-C |
| Hg38 | 4-499391-G-A | 4-499456-G-T | 4-508901-G-A | 4-516087-T-G | 4-521842-G-A | 4-523878-G-A | 4-533870-T-TA | 4-533983-T-C |
| **ACMG classification** | **Likely pathogenic** | **Pathogenic** | **VUS** | **Likely pathogenic** | **Pathogenic** | **Pathogenic** | **Likely pathogenic** | **Likely pathogenic** |
| **Criteria used** | **PM2, PM3, PS3_supp, PP4** | **PM2, PVS1, PM3** | **PM3, PS3_supp, PS4_Supp** | **PM2, PM3, PS3_supp, PP4** | **PVS1, PS4_mod, PS3_supp PM3, PP4** | **PM2, PVS1, PM3** | **PM2, PVS1** | **PM2, PVS1** |
| AC GnomadV2 (frequency) | 0 | 0 | 10* (4.0e-5) | 1 (3.98e-6) | 186 (6.58e-4) | 0 | 3 (1.2e-5) | 0 |
| AC GnomadV3  (frequency) | 0 | 0 | 6 (3.9e-5) | 2 (1.32e-5) | 107* (7.03e-4) | 0 | 0 | 0 |
| AC GnomadV4  (frequency) | 6 (4.12e-6) | 0 | 28* (1.74e-5) | 17 (1.05e-5) | 1719** (1.06e-3) | 0 | 10 (6.20e-6) | 0 |
| Amino acid residue conservation vertebrates (Basewise phyloP100way) | Chicken (5.52) | NA | Zebrafish (8.97) | Rhesus (6.47) | NA | NA | NA | NA |
| REVEL | 0.479 | NA | 0.689 | 0.360 | NA | NA | NA | NA |
| CADD/Splice AI | 28.2 | 33.0 | 25.4 | 23.7 | 42.0 | 39.0 | 32.0 | 0.98 |
| Previously reported | Novel | Novel | Tremblay-Laganière et al 2021 (1 family)  Stranneheim et al 2021 (no data) | Novel | Arteche-López et al 2021 (1 family) Tremblay-Laganière et al 2021 (1 family) | Novel | Novel | Novel |
| Segregation | In *trans* with Trp505* | In *trans* with Trp678* | In *trans* with c.2735+2T>C | In *trans* with Trp505* | Homozygosity confirmed in 2 families | In *trans* with Gly41* | - | In *trans* with Gly278Arg |
| ClinVar | VUS | NA | VUS | VUS | Conflicting | NA | P/LP | NA |
| Phenotype specificity | Emm negative |  |  | Emm negative | Emm negative |  |  |  |
| Functional evidence | Null enzyme activity in transfected HEK293 DKO cells (this paper) | - | Null enzyme activity in transfected HEK293 DKO cells (Tremblay-Laganière et al 2021) | Null enzyme activity in transfected HEK293 cells (this paper) | Reduced enzyme activity in transfected HEK293 DKO cells due to partial expression (this paper) | - | - | - |

**Supplementary Table 3: Variant classification** * 1 homozygote ** 2 homozygotes. ClinVar as of 1 Feb 2024. All variants are reported in MANE transcript NM_001127178.3. PM3 is assigned using ClinGen Sequence Variant Interpretation Recommendation for in trans Criterion (PM3) - Version 1.0 Working Group Page: https://clinicalgenome.org/working-groups/sequence-variant-interpretation/ Date Approved: May 2, 2019. NA – not applicable, VUS – variant of uncertain significance, P/LP – pathogenic/likely pathogenic, DKO – double knock out

**Other variants detected through genetic testing**

| **Individual ID** | **Gene** | **Variant Coordinates** | **Amino Acid change** | **Comment** |
| --- | --- | --- | --- | --- |
| **3:I** | DUOX2 | NM_014080.4:c.602dup | p.(Gln202Thrfs*99) | Class 1 (pathogenic); not clinically relevant – carrier of one allele in recessive disease |
| **3:I** | NPC1 | NM_000271.4:c.3182T>C | p.(Ile1061Thr) | Class 1 (pathogenic); not clinically relevant – carrier of one allele in recessive disease |
| **2:I** | BSCL2 | NM_001386028.1:c.1097C>T | p.(Thr366Ile) | Class 3 (VUS); carried by unaffected father, but not sufficient evidence to formally classify as benign |
