# Supplementary material for: Recessive Variants in PIGG Cause a Motor Neuropathy with Variable Conduction Block, Childhood Tremor, and Febrile Seizures: Expanding the Phenotype
Source: Ann Neurol. 2024 Oct 23;97(2):388–96. doi: 10.1002/ana.27113 (PMC11740278; doi:10.1002/ana.27113)
Supplement: Supplementary file 9 — Table S4. Hemagglutination results EMM antigen typing. [file ANA-97-388-s004.docx]

**Supplementary Table 4**

| PEG IAT | Individual 4:I | Individual 4:II | Positive control EMM + | Negative controle  EMM - |
| --- | --- | --- | --- | --- |
| Anti-EMM | negative | negative | 2+ | negative |
| Control reagent | negative | negative |  |  |

**Hemagglutination results of the EMM antigen typing** on the two siblings of Family 4, performed with indirect antiglobulin test with polyethylene glycol as an enhancer. The anti-EMM reagent is a polyclonal antibody derived from the plasma of on unrelated EMM negative patient. The control reagent is a plasma without antibodies. The agglutination scoring is on a scale from negative to 4+.
